# Identification of the most relevant aspects of spinal muscular atrophy (SMA) with impact on the quality of life of SMA patients and their caregivers: the PROfuture project, a qualitative study

**DOI:** 10.1186/s41687-024-00758-0

**Published:** 2024-07-24

**Authors:** Mencía de Lemus, Maria G. Cattinari, Samuel I. Pascual, Julita Medina, Mar García, Ana Magallón, María Dumont, Pablo Rebollo

**Affiliations:** 1 Fundación Atrofia Muscular Espinal (FundAME), Calle Nuria 93, 1ºC, Madrid, 28034 Spain; 2 SMA-Europe, Freiburg, Germany; 3 Committee of Advanced Therapies at the European Medicines Agency, London, UK; 4 Department of Neurology, Hospital Universitario La Paz – Madrid, Madrid, Spain; 5 Rehabilitation and Physical Unit Department, Hospital Sant Joan de Déu, Barcelona, Spain; 6 Imfisio, Madrid, Spain; 7 IQVIA, Madrid, Spain

**Keywords:** Spinal muscular atrophy, Outcome measures, Quality of life, Scales, Patient reported outcomes

## Abstract

**Background:**

SMA is a hereditary neuromuscular disease that causes progressive muscle weakness and atrophy. Several studies have shown that the burden of SMA is very high at many levels. Functional assessment tools currently used do not completely address the impact of the disease in patients’ life. The objective of this qualitative study was to identify aspects of SMA that are relevant to patients and to design items useful for assessment purposes.

**Results:**

Five focus group sessions were run during an annual SMA families meeting in Madrid, Spain. Focus groups were composed by parents of SMA type I children, sitter children type II-III, parents of sitter children type II-III, adult patients, and parents of walker children. Two trained facilitators conducted the focus groups using a semi-structured guideline to cover previously agreed topics based on the input of a Scientific and Patient Advisory Committee. The guideline was adapted for the different groups. According to what was communicated by participants, SMA entails a high burden of disease for both patients and their parents. Burden was perceived in physical, psychological, and social areas. Patient’s physical domain was the most relevant for participants, especially for parents of non-ambulant children, followed by limitations of motor scales to capture all changes, parents psychological burden, treatment expectations and patient’s psychological burden. Ten domains were the main areas identified as impacted by the disease: mobility and independence, fatigue and fatigability, infections and hospital consultations, scoliosis and contractures, vulnerability, pain, feeding, time spent in care, breathing, and sleep and rest.

**Conclusions:**

This study confirms the necessity of evaluating other aspects of the disease that are not assessed in the functional motor scale. Measures of other aspects of the disease, such as pain, fatigue, feeding, should be also considered. A patient-reported outcomes instrument measuring such aspects in a valid and reliable way would be very useful. This study generated a list of new items relevant to be systematically measured in the assessment of the impact of SMA on the patients’ everyday life.

**Supplementary Information:**

The online version contains supplementary material available at 10.1186/s41687-024-00758-0.

## Background

Spinal muscular atrophy (SMA) is a neuromuscular disease which causes progressive muscular weakness [1]. SMA encompass a spectrum of severity that range from inability to self-feed and breath independently, to progressive loss of the ability to walk, with most disease forms being highly disabling [1]. It is a rare, autosomal recessive disease that is primarily caused by a deletion or mutation of the survival motor neuron 1 (*SMN1*) gene, which encodes the SMN protein [2]. *SMN2* is a paralogous gene, which results in a transcript lacking exon 7, that can only partially compensate for *SMN1* deficiency [2, 3]. SMA patients have varying numbers of SMN2 gene copies (between 1 and 4), which significantly influence disease severity.

Currently, several motor scales are used to assess the SMA patient’s motor function. The most extensively used, of which are: for non-sitters, the Children’s Hospital of Philadelphia Infant Test of Neuromuscular disorders (CHOP INTEND) [4], aimed at children up to 38 months, and the Hammersmith Infant Neurological Examination (HINE) [5], for children younger than 24 months; for sitters, the Hammersmith Functional Motor Scale-Expanded (HFMSE) [6] or the Motor Function Measure (MFM-32) [7] and the Revised Upper Limb Module (RULM) [8]; for walkers, the 6-minute walking test (6MWT) used in combination with the HFMSE or MFM-32 and the RULM. While these scales are absolutely relevant to measure the patient’s motor status, they do not fully cover other aspects of the disease that can have a significant impact on the patient’s wellbeing [9–11], such as the ability to perform daily activities, fatigue, endurance, bulbar function (speech, respiratory function, swallowing), rest, and sleep. Besides, tools to assess the patient´s functionality, or ability to perform tasks and roles for everyday life, such as the EK2 scale or the SMA Functional Rating Scale (SMA-FRS) [12], and tools to assess the patient´s Health-Related Quality of Life (HRQoL), such as the Pediatric Quality of Life Inventory in children [13] or the Short Form-36 Health Survey (SF-36) in adults [14], are also used.

Some studies have already been conducted to broaden the understanding of the impact of SMA in both the patient and the caregiver’s life. Two studies were carried out with patients under 18 years old [9, 15]. These studies concluded that the burden of the disease is very high at many levels and that additional tools for the assessment of small changes are needed. Two other studies focused only on adult SMA population: one of them aimed at identifying the domains and symptoms that primarily impact their HRQoL [10] and the other one, at showing unmet needs that are impacting their HRQoL [16]. A fifth study focused on type II and III patients, their current clinical status, the impact of their disease on their HRQoL, and their expectations regarding treatments [11].

Other study [17] involved physicians, physical therapists, advocacy groups, and representatives from pharmaceutical companies discussing patient-reported outcomes (PROs) in SMA. They conducted a comprehensive review of existing tools to measure health-related quality of life, activities of daily living, caregiver burden, and new tools to assess aspects specifically relevant to SMA.

The emergence of new therapeutic options for SMA necessitates robust outcome measures to track disease progression in these novel treatment environments [18]. Ideally, these measures should be well-validated across a range of psychometric characteristics [18, 19]. Unfortunately, there is a current scarcity of PROMs specifically designed for SMA. Currently available SMA-specific PROMs have limitations. The Spinal Muscular Atrophy Independence Scale (SMAIS) solely focuses on independence in daily living activities for patients with SMA type 2 and 3 [20], neglecting other crucial aspects like fatigue and swallowing difficulties. The SMA Health Index (SMA-HI), while evaluating a broader range of 15 disease burden domains [21], has limited applicability due to the lack of validation in children under 8 and the absence of established responsiveness to change. This scarcity of specific PROMs for SMA highlights the critical need for tailored tools.

The present study focuses on a group of Spanish patients with SMA and the impact that the disease has on their daily life. The objective was to obtain information from SMA patients and their parents/caregivers about the burden of the disease in relation to physical, psychological, and social aspects, taking into account different age ranges and disease severity degrees. This study also sought to identify aspects of the disease that are relevant to patients, and which might be modified by treatments, other than the motor status aspects already being captured by the existing scales. The ultimate goal of the study was to identify a series of items that will enable capturing, in a systematic way, those aspects of everyday life impacted by SMA.

## Methods

### Design

The PROfuture Project is a qualitative study based on the methodology of focus groups (five groups). The study design and group composition were based on a previous literature review, including a deep review of existing outcome measures and on the previous experiences of the Spanish SMA patient organisation (FundAME). The methodology of focus groups has been previously used in patients with SMA and their caregivers [9, 15]. FundAME recruited participants from various geographic areas of Spain into its registry based on specific inclusion criteria.

The focus group sessions were run during three consecutive days, during the 2019 annual SMA families meeting held in Madrid.

### Participants

Five focus groups with the following composition:

1. Parents of SMA type I children aged 13 months-8 years (*N* = 8): Six parents of SMA type I children, aged between 13 months-5 years, and two parents of SMA children 7 and 8 years old.
2. SMA type II-III children aged 10–16 years, sitters (*N* = 8): Three 10-year-olds, one 12-year-old, two 14-year-olds, one 15-year-old and a 16-year-old.
3. Parents of SMA type II-III children aged 4 to 16 years, sitters (*N* = 10).
4. Adult patients (between 18 and 53 years old), types II and III (*N* = 7). Most had been ambulant in the past but only two were ambulant at the time of the study.
5. Parents of SMA type III children (5 children between 2 and 8 years and six children between 11 and 16 years), walkers (*N* = 11).

All participants (or their children) had been genetically diagnosed with SMA. The study was approved by the ethical committee of the Hospital Universitario La Paz, and all participants of age 10 and above (and their parents, for participants under 18 years of age) signed the informed consent form to participate in the study and to record all meetings.

### Procedures

**Fig. 1:**
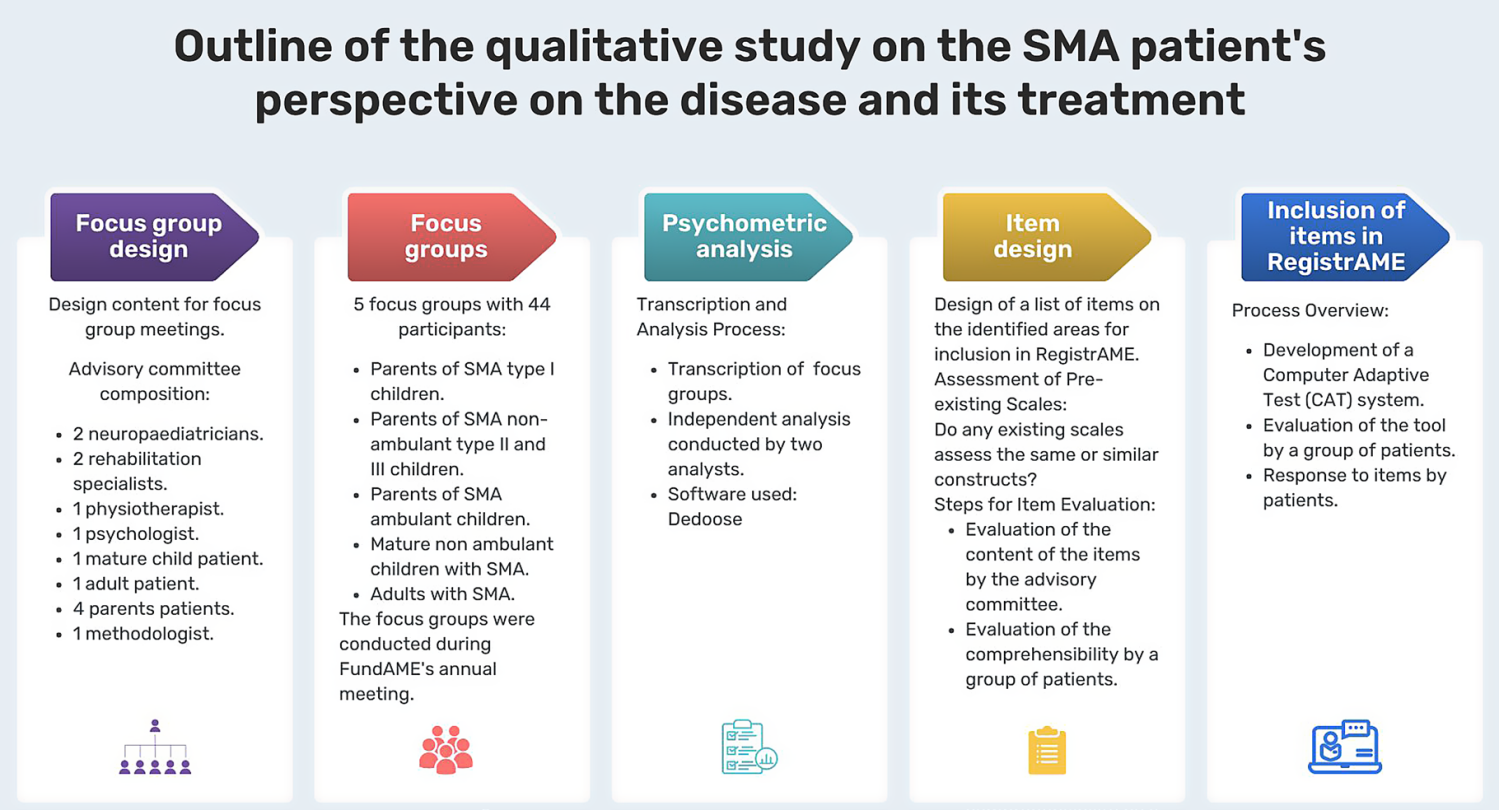
Outline of the qualitative study on the SMA patient’s perspective on the disease and its treatment

Two trained facilitators conducted the focus groups (approximately 90 min long) using a semi-structured guideline (Table 1), based on the input of a Scientific and Patient Advisory Committee (SPAC), which was adapted for the participants of each group. The composition of the SPAC was the following: six patient representatives (one adult patient and five parents of children living with SMA) and six experts on SMA (two child neurologists, two rehabilitation doctors, one physiotherapist and one psychologist), all of them with extensive SMA experience, plus a consultant with extensive experience in conducting focus groups. The SPAC participated in the development of the scripts for the focus groups, as well as in the design and revision of items. Each session was audio-recorded and transcribed for analysis. Overall study flow is presented in the Fig. 1.

**Table 1:** Semi-structured guideline for the focal groups

1. Disease-related symptoms that affect patient’s life the most
a. Symptoms related to physical health: limited ambulation and mobility, weakness in legs, weakness in arms, weakness in hands and fingers, sleep problems, pain, gastrointestinal problems, respiratory problems.
b. Symptoms related to psychological health: stress, body image.
c. Symptoms related to social health: limitations of the social role, dissatisfaction with social life.
2. Daily life activities or bodily functions that are important to SMA patients and are not included in the commonly used functional questionnaires.
a. Activities that the patients cannot do but are very important to them.
b. Activities that the patients can do and are very important to them.
3. Expectations about treatments for SMA
a. Activities important to be kept/maintained with treatment.
b. Activities important to be improved with treatment.
4. Evaluation of the commonly used functional scales as a means to assess the burden of the disease in the SMA patient’s life.
a. Do the scales reflect the functional situation of the patient?
b. Do the scales include the functional capacities that are important in the patient’s life?
c. Do changes in the scoring accurately reflect changes in real life?
d. Are the different functional capacities equally valuated to assess the patient’s evolution?

### Data analysis

**Fig. 2:**
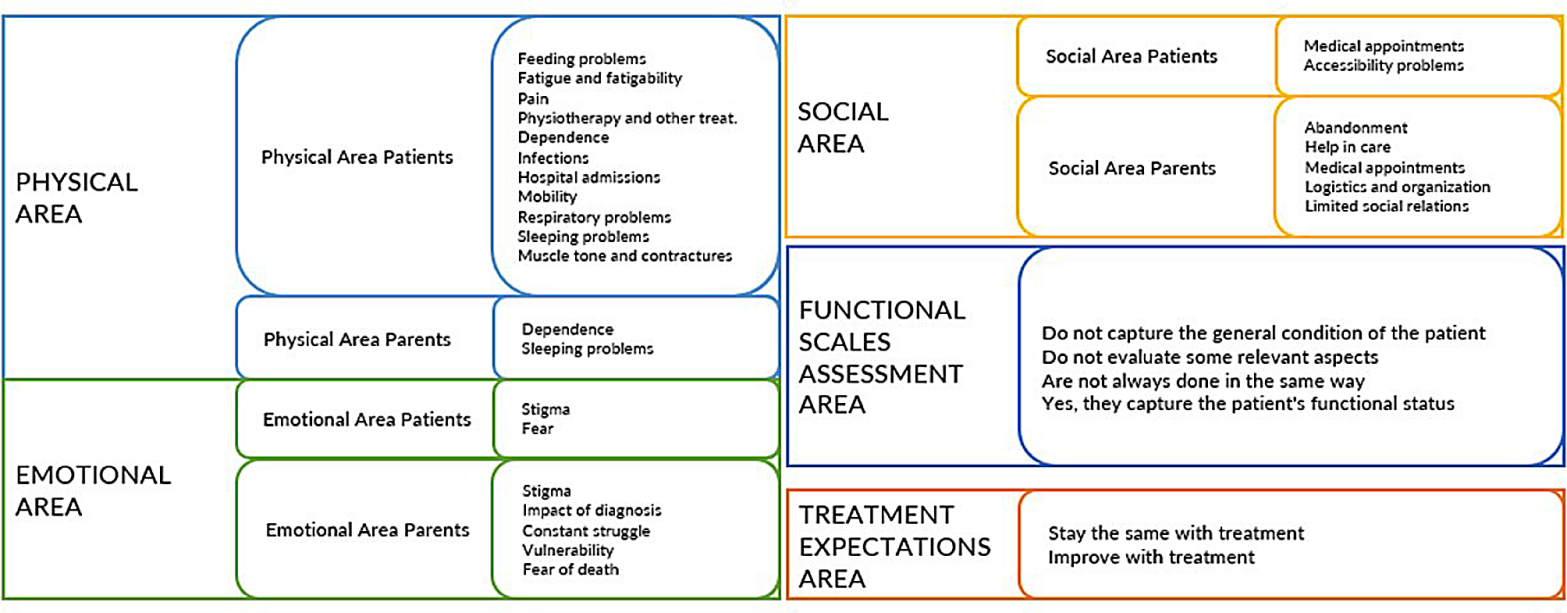
The final list of codes identified by the analysts and used in the study

The transcribed records were analysed by the Dedoose software [22, 23] to identify the relevant topics that appeared in each focus group. Two independent analysts read the transcriptions to obtain the main concepts and created a list of codes and definitions that were tested in the transcriptions; the initial list of codes was re-assessed to include additional codes. The final list of codes identified by the analysts and used in the study can be seen in Fig. 2.

From these data, general comments and experiences were obtained that were further evaluated by the SPAC, to design a list of items that captures the burden of the disease on the patients’ HRQoL, and complements the information already being captured through the existing motor, functional and HRQoL scales.

## Results

### Parents of SMA type I children

The meeting lasted 2.4 h. This group gave special importance to children’s physical domain (67 comments), parents’ psychological domain (66 comments), own experience with passing the scales (28 comments), and parents’ social domain (23 comments).

Within the children’s physical status, the most important factors were mobility and breathing, followed by muscle weakness and feeding. Parents expressed their concerns on their children breathing adequately and the risks related to respiratory infections.

The psychological burden for SMA I parents was very significant. The time dedicated to the children medical, and therapists’ appointments was pointed out as being also very significant. The parents´ social domain was very important, especially in relation to time dedicated to the multidisciplinary care of their children and the need to find help for their everyday cares.

Parents discussed the treatment-related improvement, such as better physical endurance, stiffness reduction, better school performance or gaining new abilities (such as cutting out paper). It was acknowledged that any improvement due to treatment, even lack of disease progression, had a positive impact.

Parents agreed that current functional scales are clinically relevant but inadequate in capturing the complete patient status, as they do not include crucial aspects for children and cannot assess minor changes.

### SMA type II-III children aged 10–16, non-ambulant

The meeting lasted 1.5 h. The most relevant domain was patient’s physical domain (60 comments), followed by patient’s social domain (39 comments), scales evaluation (33 comments), and treatment expectations (26 comments).

Mobility was the most important issue within the patient physical domain, almost always related to the lack of independence it causes, and the lack of autonomy for self-grooming and toileting. Being wheelchair user was generally well accepted although physical barriers at some places where mentioned as a problem. Pain was reported as an important issue, that also impacted their physical therapy sessions. The lack of ability to turn in bed and how this impacted sleep was highlighted. Other issues mentioned were vulnerability with regards to chest infections, their need for help to eat and the fear on choking on food.

Within the patient´s social domain, there were two major issues: Time spent on medical and physiotherapy appointments, and accessibility problems, since they limit their ability to join plans with their friends.

Almost all of these patients were receiving treatment, and the most important treatment-related factor for them was function improvement.

Regarding the scales, they were very critical establishing that they do not reflect important aspects of the benefit of treatments or that they do not assess other aspects that are very relevant for their daily life activities.

A list of important domains for children was designed in this group (Table 2).

**Table 2:** List of important domains designed by group 2 (SMA type II-III children aged 10–16, non-ambulant)

**Great impact**	
Physical	Not being able to transfer from the wheelchair or to use the stairs Not being able to go to the toilet alone or get dressed alone Not being able to turn in bed
	Hip pain
Psychological	Feeling of people’s disapproval
	Being treated like a child,
	Feeling they´re conditioning their friend’s and their family’s life
Social	Not being able to go to class due to medical appointments
	Feeling alone
**Medium impact**	
Physical	Not being able to shower alone Not being able to pick up things from the floor
	Pain caused by splints
Social	Not being able to do things with friends.
**Low impact**	
Physical	Not being able to raise arms all the way Not being able to do sports
Social	Lack of adaptation of public transit
	Not being able to go to the pool or beach alone
	Not being able to use funfair attractions

### Parents of SMA type II-III children, non-ambulant

This meeting lasted 1.4 h. The most important domain was the parent’s psychological domain (41 comments), followed by treatment expectations and patient’s physical domain (28 and 26 comments, respectively), and the last domain was the patient’s psychological wellbeing (23 comments).

As for the patient´s physical domain, pain, was considered very important.

In the psychological domain, some of the issues that worried parents were disease progression, the future, the adolescence period, and scoliosis. They also reported fear of the independence of their children being tied to an important economic burden and the risk of accidents that could be fatal for them. Regarding the psycho-emotional impact of the children, parents reported a “wish of normality” for their children and them being able to cope with complicated situations linked to their disability. Emotional vulnerability of the children, frustration and coping with academic challenges were also highlighted. Regarding daily life activities, parents agreed to report pain as the most important aspect affecting their children’s quality of life.

The effect of treatment was generally evaluated as positive and patient stabilization, as a success.

They agreed with the scales but commented on additional questions that should be included to assess the real patient status, such as becoming faster in doing daily life activities and a reduction in infections and fatigue.

### Adult patients (older than 16 years old), ambulant or non-ambulant

The meeting lasted 1.6 h. As in other groups, the most important domain was the patient’s physical domain (29 comments), followed by psychological domain (20 comments), and treatment expectations (18 comments).

Mobility was the main issue of the physical domain and losing the ability to walk had been devastating, regardless of the age at which it occurred, to the non-walkers. The severity of disability within the group was very broad with some participants never being able to walk while others only reported problems with climbing stairs or walking long distances, showing therefore, a good mixture of experiences. In the psychological domain, fears about the future and the disease progression were common, as well as fear of not being able to complete full working hours due to fatigue. Stigma associated to disability was also pointed out as important. In the social domain, the most important factor was accessibility limitations due to wheelchair use. This has a large impact in their social, as well as, professional life.

Regarding treatment expectations, stabilisation of their present level of independence was important, namely maintaining enough upper limb mobility to preserve basic abilities for their daily life. They expect to maintain their functional capacity with treatment and to improve in some functions.

This group was in line with all the other groups regarding the scales. They noted that some important aspects to be evaluated are sleeping well, breathing well, having less fatigue, having less pain, and finger mobility to use the phone or computer.

### Parents of SMA type III, ambulant children (*N* = 11)

The meeting lasted 1.9 h. The majority of the concerns were about treatment expectations and the use of scales to assess the child’s evolution (35 comments each one). Regarding the impact of SMA in the patient or parent’s life, the most commented was the patient’s physical domain (29 comments), followed by patient’s psychological domain (18 comments) and parent’s psychological domain (16 comments).

Parents considered the children´s physical domain as the most affected by the disease, and they greatly valued the children´s ability to be independent in some daily tasks, such as toileting. Mobility problems had great importance, since, although able to walk, these patients still have problems of dependence and fatigue, and children were reported to lack strength.

The disease´s impact on the psychological domain was equally high in children and parents. Parents commented on the strategies their children have to cope with the disease and how important it is for children to be accepted by their peers (emotional problems are sometimes derived from peer rejection). The psychological impact of SMA on parents comes mainly from a feeling of constant struggle, and from fear of disease progression or death.

Regarding treatments, parents were enthusiastic about the possibility of stabilizing the disease and improving some functions.

The evaluation of the scales reinforced the idea that they are not capturing all the important aspects of the patient’s life.

### Overall analysis

Analysing the results of all focus groups together, the patient’s physical domain received the largest attention, followed by the limitations of motor scales to capture all changes, parent’s psychological burden, treatment expectations and patient’s psychological burden.

Each focus group gave different weighting to the different domains, and an analysis according to group following the established codes was carried out (Table 3).

**Table 3:** Number of times that the main domain codes were assigned in the transcriptions of the meetings

	Group 1 Parents of SMA type I children	Group 2 Non-ambulant mature children	Group 3 Parents of non-ambulant children	Group 4 Ambulant and non-ambulant adults	Group 5 Parents of ambulant children	Total
**Physical domain**						
*Patient’s physical domain*	67	60	26	29	29	211
Feeding	11	5	2	0	1	19
Fatigue	5	5	3	6	9	28
Pain	0	9	10	1	0	20
Physiotherapist	8	4	0	1	0	13
Independence	0	0	0	9	11	20
Infections	7	2	1	0	0	10
Hospital admissions	4	5	0	0	0	9
Mobility	14	25	0	11	14	64
Respiration	13	2	1	0	3	19
Sleep	1	7	3	1	0	12
Muscle tone	12	0	4	0	0	16
*Parent’s physical domain*	11	4	8	-	5	28
Constant dependence	5	1	6	-	5	17
Sleep	8	4	2	-	0	14
**Psychological domain**						
*Patient’s psyc. domain*	7	22	23	20	18	90
Stigma	0	11	1	2	3	17
Fears	0	0	0	9	2	11
*Parent’s psyc. domain*	66	0	41	-	16	123
Stigma	6	0	3	-	2	11
Diagnosis impact	14	0	4	-	4	11
Constant struggle	15	0	2	-	5	22
Fears	20	0	18	-	4	42
Death	8	0	1	-	3	12
**Social domain**						
*Patient’s social domain*	4	39	10	11	8	72
Medical appointments	0	11	0	0	0	11
Accessibility problems	0	18	0	9	2	29
*Parent’s social domain*	23	2	6	-	1	32
Abandonment	3	0	0	-	0	3
Help with self-care	9	0	0	-	1	10
Medical appointments	6	0	0	-	0	6
Organization and logistics	6	2	0	-	0	8
Limited relationships	6	0	0	-	0	6
**New treatment expectations domain**						
*Treatment expectations*	16	26	28	18	35	123
Stay the same with treatment	5	2	6	6	10	29
Improve with treatment	7	10	12	4	10	43
**Functional scales evaluation domain**						
*Scales evaluation*	28	33	21	11	35	128
Do not capture general condition of the patient	17	16	6	3	14	56
Do not evaluate some relevant aspects	11	9	5	7	9	41
Not always done the same way	8	5	9	2	11	35
Capture the functional state of the patient	2	3	0	0	1	6

- Parents of adult patients did not participate

The patient’s physical domain was the most relevant, especially as perceived by parents of non-ambulant children (types 1–3). Within the physical domain, pain was important for non-ambulant types 2 and 3 children and their parents. Lack of independence was also perceived as very important in most groups but mainly in ambulant patients. Frequency of hospitalisation was considered relevant for SMA type I children, since it disrupts normal school and social life. Mobility was the most referred topic within all the groups, followed by fatigue and fatigability, which, according to all groups, are not being systematically covered and would be positive to have them assessed. Both, patients and parents, valued the reduction in fatigue and fatigability as one of the main gains of new treatments. Medical and therapist appointments were reported to have a great impact on patients’ and parents’ quality of life, due to the need to fit them on everyday life. Respiratory infections and the possibility of getting them have a great negative impact on patients and parents. The major concerns of parents regarding their own physical domain were sleeping problems and exhaustion.

In the psychological domain, the disease has great impact on the parents´ psychological and emotional health. Although this area was the third in importance, according to the number of codified comments, authors decided to exclude it from the final list of items because other tests already address it. Additional topics that were important to parents were the impact of diagnosis, stigma and fear of death. For non-ambulant children, the main concern was stigma, particularly regarding other people’s opinions on wheelchairs. Adult patients worried about the future, disease evolution and the fear of not to being able to conduct a normal daily life, while parents worried about the feeling of the great vulnerability of their children, who can suffer important health damages from minor events.

The impact in the social domain was especially relevant in adults and in non-ambulant children and their parents. Interference of medical and therapy appointments with school life had a lot of comments in non-ambulant children, for whom accessibility problems were also relevant, since they have a direct impact on school and social life. It seems relevant to analyse how the patient’s functional situation and its evolution impacts in a normal social life at school and work or in leisure activities.

Regarding treatment expectations, remaining stable was highly valued in all groups. In addition to remaining stable, the discovery of a new treatment able to improve their life would be highly appreciated. Priorities differed by group: Maintaining or improving respiration and feeding (parents of SMA type I children and of non-ambulant children), using the toilet and taking a shower independently, and writing for long periods of time (non-ambulant children), resistance to fatigue and better functional capacity in the upper limbs (parents of non-ambulant children), maintaining or improving independence level (SMA adults), and maintaining the functional capacity required to have an independent life (parents of ambulant SMA children).

### Item design

Analysing the results of these focus groups, it was concluded that the most important areas to evaluate the burden of SMA in the patient’s life were:

1. Fatigue, fatigability and strength
2. Pain
3. Scoliosis, contractures and hip dislocation
4. Feeding
5. Breathing and voice
6. Sleep and rest
7. Vulnerability
8. Infections and hospital admissions
9. Time spent in care
10. Mobility and independence

Stigma and some of the fears the patients reported will not be included in the resulting questionnaire as there are existing outcome measures that already address them sufficiently. After evaluating the data, comments and experiences shared at the focus groups, the SPAC designed new items with improved definitions of the relevant topics identified. A comprehensive and appropriate assessment of these items was performed by a group of 22 expert patients and parents in two rounds: the first round using paper-based questionnaires and the second one using Computerized Adaptive Testing (CAT) technology. The final list (Appendix 1) of items has been integrated into the Spanish Registry of SMA Patients-RegistrAME, to conduct a formal psychometric validation.

## Discussion

PROs are essential for capturing the patient´s perspective about the impact of disease, its progression, and the response to treatment, especially in chronic conditions such as SMA, because the perspective of patients is essential to determine which symptoms and health outcomes are relevant [24, 25]. A good PRO shows the aspects that patients consider most relevant to improve their condition and their lives [26]. Full patient involvement in the development of PROs is essential to capture the real patient perspective.

Current tools used to assess people living with SMA are very useful in the clinical practice to assess the motor function status of patients but do not completely assess other dimensions of the disease and might not be sufficient to evaluate all the impact that the new available treatments may have on a patient [27]. Furthermore, several studies have shown that existing measures exhibit floor and ceiling effects for motor function in patients at both ends of the disability spectrum [28]. In addition to the existing tools, it is important to assess how the disease is impacting on what can be more relevant to the patient´s daily life.

The present study organized focus groups with Spanish SMA patients and parents/caregivers to qualitatively assess the impact of the disease on physical, psychological and social aspects of their lives.

The patient’s physical aspects, specially concerning motor function, was considered as the most impacted by the disease, and mobility was the most referred topic within all the groups, in agreement with previous studies [9, 10]. Previous studies have shown that mobility or walking limitations [10], small changes in motor function [9], being able to use the restrooms alone, self-feeding, washing themselves, and performing transfers on their own [11] have an important impact on HRQoL.

The current study also showed a great psychological impact in patients and their families, in agreement with a previous study carried out with focus groups in the clinical setting of the United States, which showed similar psychosocial thematic areas with great impact on patients and their families, such as confronting premature death, fearing the loss of functional ability, loss of sleep and stress, stigma, limitations on social activities, and independence [15].

Regarding the expectations for new treatments, remaining stable was highly valued by the patients and their parents, as it always is for new treatments in chronic, degenerative and incapacitating diseases, in which it is very important that treatments prevent disease progression. This fact has been confirmed by comments in all the focus groups of this study. A PRO measure designed to assess patient treatment satisfaction in chronic diseases, the Treatment Satisfaction Questionnaire for Medication [29] German version 1.4 (TSQM-1.4©), was reported as useful to assess patients’ satisfaction with the first approved disease-modifying therapy for SMA [30], and its first question asks about satisfaction with the ability of the medication not only to treat but also to prevent the condition.

Patients valued highly the current motor function scales although they agreed that these scales do not address some aspects of the disease that are relevant to patients and caregivers, which they would find important to assess when evaluating response to treatments. These would include fatigue and fatigability, pain, daily life activities (such as feeding, toileting, dressing, using a computer or cell phone autonomously) respiratory capacity and sleeping. Both parents and patients would be happy having these aspects covered systematically during the patient’s assessments. A previous study, carried out with the focus group approach, also concluded that the burden of the disease is very high at many levels, and that additional tools for the assessment of small changes are highly needed [9]. The McGraw study reported that patients were not fully satisfied with how the scales assessed their whole functional state and, with how they captured small changes, and agreed that scales should include other important features, such as the ability to perform daily activities, respiratory function, swallowing, fatigue, and endurance [9].

Some of the items designed based on the results of this study, such as pain and vulnerability, are not traditionally considered in the evaluation of SMA patients. Vulnerability could be defined as the susceptibility and risk that contribute to having an increased possibility of being hurt physically, emotionally or mentally [31, 32]; it is a state of risk [31]. The physical vulnerability of patients is in many cases accompanied by emotional vulnerability; the diagnosis of a severe progressive chronic condition or the prospect of death can cause fear, anger, and despair [31]. In fact, in the current study, some patients and families expressed their concern about the risk of dying due to daily situations, such as choking when swallowing or getting a serious respiratory infection after minimal social exposure. Three areas of vulnerability were mainly identified: risks during feeding, susceptibility to respiratory infections, and the risk of losing and not being able to regain a safe body posture (falling, bending.). This implies that activities that the patient could perform independently (e.g., eating, staying alone) are not done because the risk of an accident happening is too high. Likewise, patients may feel forced to restrict their social life, for fear of potential events happening (for example limiting home visits to avoid respiratory infection). This leads to a greater burden on caregivers and a greater dependency in the case of patients.

We have identified areas and items important to assess the burden of SMA in the patient’s life, which constitute a patient relevant dataset. This dataset might, down the road, give rise to a reliable PRO, as patients were involved in identifying the outcomes and generating the items, as well as checking their comprehensibility and content validity [25]. This dataset (Appendix 1) has been included in the FundAME’s registry (RegistrAME). Through the data gathered with the Registry, the selected items will be validated and will serve as a basis to draw a new PRO. A few of these items (regarding fatigability, breathing and voice, sleep and rest, and vulnerability) have been included in a set of patient- and caregiver-oriented measurements to assess health outcomes in SMA, which is currently being validated in a study [20]; however, the full set of items resulting from this qualitative study has not been previously published.

Currently there are only 2 PROMS specifically developed for SMA. Among these, the SMAIS assesses independence in daily living activities for individuals with SMA type 2 or 3 [20], but it doesn’t consider other important aspects of the disease burden identified in our present study. On the other hand, the SMA-HI has proven to be a disease-specific, relevant, valid, and reliable outcome measure to assess multifaceted patient-reported disease burden in older children, teenagers, and adults with SMA [21, 33]. The present study results in a dataset distinct from SMA-HI utilizing focus group methodology with substantial patient representation and engagement throughout all phases of item development. However, the SMA-HI may require further validation of its responsiveness to change, and its restricted accessibility as a paid scale may limit its widespread adoption and utility in research and clinical practice. Furthermore, the resulting articles from this study will be made available as open access.

The most commonly used PROM for adults with SMA is the SMA Functional Rating Scale (SMAFRS), derived from the Amyotrophic Lateral Sclerosis Functional Rating Scale (ALSFRS). While the original SMAFRS lacked domains for swallowing and respiratory functions [18], a recent version has addressed this gap. Although the updated version shows stability and unidimensionality [34], larger-scale studies are needed for further validation [34]. Importantly, the new SMAFRS still excludes key areas identified as relevant in our study, such as fatigue, pain, and speech. Additionally, similar to the original version, it does not cover the pediatric population.

Patient response to current treatments is not uniform [35]. It is important to have measurement tools that capture minimal change that is clinically meaningful, especially if decisions of treatment discontinuation can be made based on disease worsening or inability to capture improvement [36]. The current pharmacoclinical treatment protocol in Spain evaluates the patient response to treatment based on motor function, by means of the HINE scale Sect. 2, CHOP INTEND, HFMSE, RULM and 6MWT and on respiratory function, by changes in ventilation. The mentioned tools are used for the patient assessment by the physical therapist and the physician, but the physician´s opinion and general patient assessment, as well as the patient´s perception on his own health status must also be considered. The current study shows that there are other relevant issues considered by the patients as having a great impact in their lives, which should be evaluated.

The main limitation of the study is the qualitative methodology, which is somewhat subjective, and the small size of the studied groups. However, this methodology has been used in previous SMA studies [9–11, 15], and it is known that qualitative research is an important component in PRO instrument development [26].

## Conclusions

This study provides very useful data to include the perspective of the patient in the design of new outcomes for SMA patients. The study results agree with the literature, highlighting the motor limitations as the most important aspects of the burden of the disease. Thus, the study confirms that the scales of motor function and disability are very useful in the assessment of disease burden, although measures of other aspects of the disease, such as pain, fatigue, swallowing, respiration, in addition to social and psychological impact, should be added in the patient evaluation, and a PRO instrument measuring such aspects in a valid and reliable way would be very useful.

The outcome is a list of items of the areas considered as relevant to be systematically measured in the assessment of the impact of SMA on the patients’ everyday life.

### Electronic supplementary material

Below is the link to the electronic supplementary material.

Supplementary Material 1

## Data Availability

The datasets used and/or analysed during the current study are available from the corresponding author on reasonable request.

## References

[1] D’Amico A, Mercuri E, Tiziano FD, Bertini E (2011) Spinal muscular atrophy. Orphanet J Rare Dis 6:71 22047105 10.1186/1750-1172-6-71 PMC3231874

[2] Lefebvre S, Burglen L, Reboullet S, Clermont O, Burlet P, Viollet L et al (1995) Identification and characterization of a spinal muscular atrophy-determining gene. Cell 80(1):155–165 7813012 10.1016/0092-8674(95)90460-3

[3] Talbot K, Tizzano EF (2017) The clinical landscape for SMA in a new therapeutic era. Gene Ther 24(9):529–533 28644430 10.1038/gt.2017.52 PMC5628264

[4] Glanzman AM, Mazzone E, Main M, Pelliccioni M, Wood J, Swoboda KJ et al (2010) The children’s hospital of Philadelphia Infant Test of Neuromuscular disorders (CHOP INTEND): test development and reliability. Neuromuscul Disord 20(3):155–161 20074952 10.1016/j.nmd.2009.11.014 PMC3260046

[5] Haataja L, Mercuri E, Regev R, Cowan F, Rutherford M, Dubowitz V et al (1999) Optimality score for the neurologic examination of the infant at 12 and 18 months of age. J Pediatr 135(2 Pt 1):153–161 10431108 10.1016/S0022-3476(99)70016-8

[6] O’Hagen JM, Glanzman AM, McDermott MP, Ryan PA, Flickinger J, Quigley J et al (2007) An expanded version of the Hammersmith Functional Motor Scale for SMA II and III patients. Neuromuscul Disord 17:693–697 17658255 10.1016/j.nmd.2007.05.009

[7] Berard C, Payan C, Hodgkinson I, Fermanian J, Group MFMCS (2005) A motor function measure for neuromuscular diseases. Construction and validation study. Neuromuscul Disord 15(7):463–470 16106528 10.1016/j.nmd.2005.03.004

[8] Mazzone ES, Mayhew A, Montes J, Ramsey D, Fanelli L, Young SD et al (2017) Revised upper limb module for spinal muscular atrophy: development of a new module. Muscle Nerve 55(6):869–874 27701745 10.1002/mus.25430

[9] McGraw S, Qian Y, Henne J, Jarecki J, Hobby K, Yeh WS (2017) A qualitative study of perceptions of meaningful change in spinal muscular atrophy. BMC Neurol 17(1):68 28376816 10.1186/s12883-017-0853-y PMC5381033

[10] Mongiovi P, Dilek N, Garland C, Hunter M, Kissel JT, Luebbe E et al (2018) Patient reported impact of symptoms in spinal muscular atrophy (PRISM-SMA). Neurology 91(13):e1206–e14 30143566 10.1212/WNL.0000000000006241 PMC6161547

[11] Rouault F, Christie-Brown V, Broekgaarden R, Gusset N, Henderson D, Marczuk P et al (2017) Disease impact on general well-being and therapeutic expectations of European type II and type III spinal muscular atrophy patients. Neuromuscul Disord 27(5):428–438 28237437 10.1016/j.nmd.2017.01.018

[12] Sansone VA, Walter MC, Attarian S, Delstanche S, Mercuri E, Lochmuller H et al (2020) Measuring outcomes in adults with spinal muscular atrophy - challenges and future directions - meeting report. J Neuromuscul Dis 7(4):523–534 32538864 10.3233/JND-200534

[13] Vaidya S, Boes S (2018) Measuring quality of life in children with spinal muscular atrophy: a systematic literature review. Qual Life Res 27(12):3087–3094 30043243 10.1007/s11136-018-1945-x

[14] Kruitwagen-Van Reenen ET, Wadman RI, Visser-Meily JM, van den Berg LH, Schroder C, van der Pol WL (2016) Correlates of health-related quality of life in adult patients with spinal muscular atrophy. Muscle Nerve 54(5):850–855 27074445 10.1002/mus.25148

[15] Qian Y, McGraw S, Henne J, Jarecki J, Hobby K, Yeh WS (2015) Understanding the experiences and needs of individuals with spinal muscular atrophy and their parents: a qualitative study. BMC Neurol 15:217 26499462 10.1186/s12883-015-0473-3 PMC4619513

[16] Walter MC, Chiriboga C, Duong T, Goemans N, Mayhew A, Ouillade L et al (2021) Improving care and empowering adults living with SMA: a call to action in the New Treatment Era. J Neuromuscul Dis 8(4):543–551 33646175 10.3233/JND-200611 PMC8385518

[17] Mercuri E, Messina S, Montes J, Muntoni F, Sansone VA, all p, et al. (2020) Patient and parent oriented tools to assess health-related quality of life, activity of daily living and caregiver burden in SMA. Rome, 13 July 2019. Neuromuscul Disord 30(5):431–436 10.1016/j.nmd.2020.02.019 32386743

[18] Slayter J, Casey L, O’Connell C (2023) Patient reported outcome measures in adult spinal muscular atrophy: a scoping review and graphical visualization of the evidence. J Neuromuscul Dis 1 de enero de 10(2):239–250 10.3233/JND-221595 PMC10041426 36530090

[19] Voet N, Pater R, Garmendia J, Sistiaga A, Labayru G, Gallais B et al (2024) Patient-reported outcome measures in Neuromuscular Diseases: a scoping review. J Neuromuscul Dis 19 de marzo de;1–11 10.3233/JND-240003 PMC11091642 38517800

[20] Vázquez-Costa JF, Branas-Pampillón M, Medina-Cantillo J et al (2023) Validation of a set of instruments to assess patient- and caregiver-oriented measurements in spinal muscular atrophy: results of the SMA-TOOL study. Neurol Therapy 12(1):89–105 10.1007/s40120-022-00411-2 PMC9837344 36269538

[21] Zizzi CE, Luebbe E, Mongiovi P, Hunter M, Dilek N, Garland C et al (2021) The spinal muscular Atrophy Health Index: a novel outcome for measuring how a patient feels and functions. Muscle Nerve 63(6):837–844 33711174 10.1002/mus.27223

[22] Lieber E, Weisner T (2010) Meeting the practical challenges of mixed methods research. In: Tashakkori A, Teddlie C (eds) SAGE handbook of mixed methods in social and behavioral research. SAGE Publications, Inc, pp 559–580

[23] Lieber E, Weisner T (2012) Dedoose. https://www.dedoose.com

[24] Haywood KL (2006) Patient-reported outcome I: measuring what matters in musculoskeletal care. Musculoskelet Care 4(4):187–203 10.1002/msc.94 17117444

[25] Wiering B, de Boer D, Delnoij D (2017) Patient involvement in the development of patient-reported outcome measures: a scoping review. Health Expect 20(1):11–23 26889874 10.1111/hex.12442 PMC5217930

[26] Turner RR, Quittner AL, Parasuraman BM, Kallich JD, Cleeland CS, Mayo FDAP-ROCMG (2007) Patient-reported outcomes: instrument development and selection issues. Value Health 10 Suppl 2:S86–93 17995478 10.1111/j.1524-4733.2007.00271.x

[27] Pierzchlewicz K, Kepa I, Podogrodzki J, Kotulska K (2021) Spinal muscular atrophy: the use of functional motor scales in the era of disease-modifying treatment. Child Neurol Open 8:2329048X211008725 33997096 10.1177/2329048X211008725 PMC8107939

[28] Vázquez-Costa JF, Povedano M, Nascimiento‐Osorio AE, Moreno Escribano A, Kapetanovic Garcia S, Dominguez R et al (2022) Validation of motor and functional scales for the evaluation of adult patients with 5q spinal muscular atrophy. Eur J Neurol Diciembre De 29(12):3666–3675 10.1111/ene.15542 PMC9826246 36047967

[29] Atkinson MJ, Sinha A, Hass SL, Colman SS, Kumar RN, Brod M et al (2004) Validation of a general measure of treatment satisfaction, the treatment satisfaction questionnaire for medication (TSQM), using a national panel study of chronic disease. Health Qual Life Outcomes 2:12 14987333 10.1186/1477-7525-2-12 PMC398419

[30] Osmanovic A, Ranxha G, Kumpe M, Wurster CD, Stolte B, Cordts I et al (2021) Treatment satisfaction in 5q-spinal muscular atrophy under nusinersen therapy. Ther Adv Neurol Disord 14:1756286421998902 33747131 10.1177/1756286421998902 PMC7940734

[31] Boldt J (2019) The concept of vulnerability in medical ethics and philosophy. Philos Ethics Humanit Med 14(1):6 30975177 10.1186/s13010-019-0075-6 PMC6458617

[32] Cambridge Dictionary: Cambridge University Press (2022) [https://dictionary.cambridge.org/dictionary/english/vulnerability]

[33] Sansone VA, Pirola A, Lizio A, Greco LC, Coratti G, Casiraghi J et al (2021) The spinal muscular Atrophy Health Index: Italian validation of a disease-specific outcome measure. Neuromuscul Disord 31(5):409–418 33773884 10.1016/j.nmd.2021.02.006

[34] Sadjadi R, Kelly K, Glanzman AM, Montes J, Linsenmayer M, Tellez M et al (2023) Psychometric evaluation of modified spinal muscular atrophy functional rating scale (SMAFRS) in adult patients using Rasch analysis. Muscle Nerve marzo de 67(3):239–243 10.1002/mus.27785 36605016

[35] Tizzano EF (2019) Treating neonatal spinal muscular atrophy: a 21st century success story? Early Hum Dev 138:104851 31604576 10.1016/j.earlhumdev.2019.104851

[36] [Pharmacoclinical protocol for treatment of patients with spinal muscular atrophy 5q with the drug spinraza] Ministerio de Sanidad, Servicios Sociales e Igualdad; 2018 [cited 2021 November 11th]. http://genm.sen.es/attachments/article/164/protocolo_tratamiento_nusinersen_ame.pdf

