# Supplementary material for: Identification of the most relevant aspects of spinal muscular atrophy (SMA) with impact on the quality of life of SMA patients and their caregivers: the PROfuture project, a qualitative study
Source: J Patient Rep Outcomes. 2024 Jul 24;8:78. doi: 10.1186/s41687-024-00758-0 (PMC11266339; doi:10.1186/s41687-024-00758-0)
Supplement: Supplementary file 1 — Supplementary Material 1 [file 41687_2024_758_MOESM1_ESM.docx]

**Appendix 1. PROFUTURE Dataset**

***Note: This is a translation of the Spanish version of the questionnaire and it is not a validated version.**

**All questions are shown. For the online questionnaire, some questions will show according to previous answers.**

1. **Fatigue and Fatigability**

**1.1 During the last month…**

Have you needed to rest often during the day or for long periods because you felt tired?

Have you needed to choose your activities during the day to be able to do what you wanted?

Have you had problems to maintain your posture during the day because you felt tired?

Have there been activities you were able to do in the morning but could not do in the afternoon or evening/night (you ran out of energy as the day went on)?

If you made a bigger exertion than usual, tiredness lasted until the next

**ANSWERS**: Never, Almost never, Sometimes, Almost Always, Always

**1.2 During the last month, how much difficulty have you had in successfully completing the following activities that included repetitive or continuous movements?**

Taking notes on paper

Sending text messages

Combing your hair

Brushing your teeth.

Eating by yourself

Use of joystick

Moving in a manual wheelchair

**ANSWERS:**  Impossible, Very difficult, Difficult, Somewhat easy, Easy, Not applicable

**1.3 During the last month, have you been able to do the following movements several times with the same strength and speed?...**

For non-walking patients:

Sit up straight in the chair when your back is supported

Maintain the position of your head

Repeat specific movements when you are playing

For walking patients:

Walk up a whole flight of stairs

Keep the pace when you walk

Rise from a chair or the bed

Get in or out of a car

**ANSWERS:**  Impossible, Very difficult, Average difficulty (not easy or difficult), Easy, Not applicable

1. **Pain**

**2.1 Over the past month…**

I had pain

**ANSWERS**: Never, Almost never, Sometimes, Almost Always, Always

Only if the answer is any other than "never"

**2.2 Over the last month…**

Pain has limited your movements

Pain has hindered your grooming

Pain has made it difficult for you to do the exercises you need to do (stretching, standing...)

Pain has affected your school, work or home life

Pain has affected your social life

Pain has affected your sleep

Pain has altered your mood

**ANSWERS**: Never, Almost never, Sometimes, Almost Always, Always

**2.3 When you have felt pain, how long did it last?**

**ANSWERS**: A few seconds- A few minutes -A few hours -A few days (Less than a week) - More than a week

**2.4 Looking at the scale, select from the drop-down the value that represents the maximum pain you have felt during the last month.** (The value must be given by the patient himself/herself, not the parents or caregivers)

Visual Analogue Scale (Vas) For Pain Measurement

**NO PAIN AS MUCH PAIN AS POSSIBLE**

**
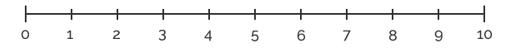
**

1. **Scoliosis, contractures and hip dislocation**

**3.1 Do you have scoliosis or have had back surgery?**

**ANSWERS**: Yes - No

Only for those answering YES, answer questions 3.2 and 3.3.

**3.2 Through the last month the scoliosis or surgery…**

has limited your movements

has made it difficult for you to maintain your posture

**ANSWERS**: Nothing - a little – Moderately- A lot – Very Much

**3.3 Do you have retractions, shortening or contractures?**

**ANSWERS**: YES – NO

Only for those answering YES to question 3.3., answer question 3.4

**3.4 Through the last month the retractions, shortenings or contractures…**

have limited your movements

have made it difficult for you to stand upright

have made hygiene difficult for you

have made it difficult for you to wear your shoes

have made it difficult for you to take care of your oral hygiene

**ANSWERS**: Nothing - a little – Moderately- A lot – Very Much

**3.5 Do you have problems with your hips (dislocation, subluxation, joint limitation or pain)?**

**ANSWERS**: YES – NO

Only if the answer is YES, answer question 3.6

**3.6 Through the last month…**

problems in the hips have limited your movements

your hips have been hurting

**ANSWERS**: Nothing - a little – Moderately- A lot – Very Much

1. **Feeding**

**4.1 Through the last month you have fed yourself…**

**ANSWERS:** Entirely by mouth - Partly by mouth - Entirely by gastric button

**If you have been fed by gastric button, answer questions 4.2 to 4.5.**

**4.2 what has your diet been like? Choose all the options that fit your situation:**

Only milk

I can eat mashed food

In continuous feeding

In fractional feeding

**4.3 I can swallow saliva**

**ANSWERS**: YES – NO

**4.4 Through the last month, how much of an appetite did you usually had?**

**ANSWERS**: Nothing - a little – Moderately- A lot – Very Much

**4.5 Through the last month, your eating difficulties have impacted your social life (you have felt uncomfortable or it has made your social life somewhat difficult)**

**ANSWERS:** Never – Almost Never – Sometimes – Most of the times – Always – Not applicable

**If you have been fed entirely by mouth, answers questions 4.6 to 4.9**

**4.6** **During the last month, at a regular meal...**

Have you have eaten any kind of food regardless of its consistency or texture (broth, steak, stew...)

Have you eaten without any help? (except for cutting steak)

Have you been able to cut a steak

Have you been able to open a yoghurt

Have you been able to open a drink can

Have you been able to open a new screw-top bottle

Have you been able to open a used screw-top bottle

Have you been able to use the spoon

**ANSWERS:** Never – Almost Never – Sometimes – Most of the times – Always – Not applicable

**4.7 During the last month, when you have drunk liquids …**

Have you needed help to bring the glass close to your mouth?

Have you needed to use a straw?

Have you needed to use a plastic cup?

Have you used a glass?

**ANSWERS:** Never – Almost Never – Sometimes – Most of the times – Always – Not applicable

**4.8 During the past month, you have taken longer to eat than before.**

**ANSWERS:** Never – Almost Never – Sometimes – Most of the times – Always – Not applicable

**4.9 During the last month, your eating difficulties have impacted your social life (you have felt uncomfortable or it has made your social life somewhat difficult)**

**ANSWERS:** Never – Almost Never – Sometimes – Most of the times – Always – Not applicable

1. **Breathing and voice**

**5.1** Can you speak?

**ANSWERS**: YES – NO – Not Applicable

**5.2 During the last month…**

Can you cough effectively (expelling mucus)?

How difficult is it for you to make yourself understood when speaking with someone you know?

How difficult is it for you to make yourself understood when speaking with someone you don’t know?

How difficult is it for you to make yourself understood when speaking by phone?

How difficult is it for you to participate in group conversations?

How difficult is it for you to make yourself understood with background noise?

**ANSWERS:** I have no difficulty – It’s a bit difficult – It’s difficult – It’s very difficult – It’s impossible – Not applicable

**5.3 Have you had changes in the quality of your voice apparently caused by secretions in your throat (wet voice)?**

**ANSWERS:** Yes – No – Sometimes – I don’t know what a wet voice is

1. **Sleep and rest**

**6.1 During the last month…**

1-Have you woken at night to ask for help to be able to move in bed?

2-If you use a ventilation machine, have you woken at night because it bothered you?

3-Have you gotten up tired after sleeping at night?

**ANSWERS:** Never – Almost Never – Sometimes – Most of the times – Always

**6.2 On the nights that you have woken, how many times do you usually wake?**

**ANSWERS:** 1 – 2-3 – More than 3

1. **Vulnerability**

**7.1 During the last month…**

How many times have you choked (when eating, speaking, because of mucus, etc.)?

**ANSWERS:** Never – Very few days – Some days – Many days – Almost every day

If the previous answer was other than “never”

**7.2 When you have choked, did you feel you couldn’t breathe?**

**ANSWERS:** Never – Very Few days – Some days – Many days – Almost every day – Not applicable

**7.3 During the last month…**

Has the risk of choking made you always eat with your usual assistant?

Has the risk of choking limited the kind of food you eat when accompanied by someone who is not your usual assistant?

Has the risk of choking limited the kind of food you can eat by yourself?

**ANSWERS:** Never – Very Few days – Some days – Many days – Almost every day – Not applicable

**7.4 During the last month, has the risk of having respiratory infections limited your school life, your social life or your visits to health centers and hospitals?**

**ANSWERS:** Never – Very Few days – Some days – Many days – Almost every day – Not applicable

**7.5 During the last month, how often have you been unable to recover an upright posture?**

**ANSWERS:** Never – Very Few days – Some days – Many days – Almost every day – Not applicable

**If the previous answer was other than “never”,**

**7.6 During the last month, have you felt at risk because you couldn’t recover your posture?**

**ANSWERS:** Never – Very Few days – Some days – Many days – Almost every day – Not applicable

**7.7 During the last month, has the risk of not being able to recover your posture led you to not being able to be alone?**

**ANSWERS:** Never – Very Few days – Some days – Many days – Almost every day – Not applicable

1. **Infections and Hospital ADMISSIONS**

**8.1 Episodes of respiratory infections during the last month**

**ANSWERS**: 0 – 1 – 2 – 3 – More than 3

**8.2** **Number of visits to the emergency department in the last 3 months**

**ANSWERS**: 0 – 1 – 2 – 3 – More than 3

**8.3** **Number of days spent in hospital or at home during the last 3 months (excluding routine treatment and follow-ups)**

**ANSWERS**: 0 – 1 – 2 – 3 to 7 – More than 7

**8.4 Referring to the days in hospital mentioned above, how many were in the intensive care unit during the last 3 months?**

**ANSWERS**: 0 – 1 – 2 – 3 to 7 – More than 7

1. **Time spent In care**

**9.1 During the last month…**

medical appointments have interfered with my attendance at school or work

the usual care (physiotherapist, speech therapist, standing, stretching, breathing exercises...) has interfered with my attendance at school, classes or work

the usual care (physiotherapist, speech therapist, standing, stretching, breathing exercises...) has interfered with my leisure/rest time/other activities

**ANSWERS:** Never – Almost Never – Sometimes – Most of the times – Always

**9.2 How many hours per week do you dedicate to your usual care (physiotherapist, speech therapist, standing, stretching, breathing exercises...) including displacements?**

**ANSWERS:** Between 0 and 20 h – 20 and 30 h – more than 30 h

**9.3 Because of your illness you spend more time than other people on routine activities. How much extra time do you need to do everyday routine activities? (compared to a person without mobility limitations)**

Dressing

Washing

Going to the toilet

Displacements (ramps, lifts, anchorages...)

Overcoming barriers at school or work

Overcoming barriers in leisure time

**ANSWERS:** Same as others – Less than 10 minutes more – 10 to 20 minutes more – 20 to 30 minutes more – More than 30 minutes more – Not applicable (e.g. due to age)

1. **Mobility and Independence**

**10.1 As an affected person, think about how much help you have needed from another person to do the following activities in the past week**

**Bathroom and hygiene**

Washing your hair

Washing your face

Washing your hands

Washing your body

Washing your teeth

Brushing your hair

Peeing

Cleaning yourself after using the toilet

**Getting dressed**

Putting on your shirt

Taking off your shirt

Putting on your jacket or windbreaker

Taking off your jacket or windbreaker

Putting on your trousers or skirt

Taking off your trousers or skirt

Putting on your socks or shoes

Taking off your socks or shoes

**Lifting or moving objects**

Picking up small objects from the table (e.g. coins or a pencil)

Picking up a large object from the table (e.g. a bottle or a one-litre carton).

Dragging a large object across the table (e.g. a bottle or a one-litre carton)

Getting something in and out of the rucksack or saddle bag

**Mobility**

Holding the head upright without a support or strap

Sitting still

Moving around your house on a manual wheelchair

Moving around your house on a powered wheelchair

Transferring to and from your wheelchair (e.g., to your bed or the bathroom)

Changing position in bed to make yourself more comfortable

Moving around your house without a manual wheelchair

Moving around your house without a powered wheelchair

Getting around by public transportation

Moving outside your house on a manual wheelchair

Moving outside your house on a powered wheelchair

Getting back up when you fall

**Tasks**

Doing household chores (e.g. tidying up, setting the table, cleaning the house, washing the dishes or doing the laundry)

Shopping (e.g. supermarket or clothes shopping)

**Other tasks**

Using a touch screen (e.g. phone or tablet)

Using the TV remote control

Using the computer mouse

Using the computer keyboard

Turning the page of a book or magazine

Writing or colouring with a pen, pencil or crayons

Opening and closing doors

Getting in and out of the car

**ANSWERS:** I didn’t need any help – I needed some help – I needed moderated help – I needed a lot of help – Someone has to do it for me – Not applicable (e.g. due to age)

**10.2 During the last month, how much difficulty have you had in carrying out the following activities?**

Going alone in the street, away from home

Being at home alone for a while

Staying in bed sleeping alone at home

**ANSWERS:** Impossible – Very difficult – difficult – Something Easy – Easy – Not Applicable (e.g. due to age)

**10.3 During the last month, have you needed assistance in your daily life?**

**ANSWERS:** Continuously – A lot – Quite – A little – Nothing

1. **Open option: “Relevant remarks to be taken into account”**
